# Bacterial metatranscriptome of dentin caries

**DOI:** 10.1080/20002297.2017.1325194

**Published:** 2017-05-30

**Authors:** Anne CR Tanner, Christine Kressirer, Lina Faller, Kristie Lake, Floyd Dewhirst, Alexis Kokarasb, Bruce Paster, Jorge Frias-Lopez

**Affiliations:** ^a^ The Forsyth Institute, Cambridge MA USA, Boston MA USA; ^b^ Harvard School Dental Medicine, Boston MA USA; ^c^ Community Dental Center, Waterville ME, USA

## Abstract

Dental caries results from altered microbial community activity in disease compared to health. Caries advanced into dentin was compared with coronal caries and caries-free sites.

Oral bacterial samples were obtained from young children, placed in RNAlater and stored frozen. mRNA was purified and sequenced on an Illumina NextSeq 500 platform. Gene sequences were aligned against taxa in HOMD and gene expression was compared between dentin and coronal caries and caries-free sites using GO terms.

There were more genes expressed in caries than caries-free sites. Coronal samples grouped together but not with dentin caries. Compared with caries-free, coronal caries species with higher gene expression included *S. mutans, Streptococcus* and *Actinomyces* whereas in dentin expressed genes mapped principally to *S. mutans* and *Scardovia wiggsiae*. Higher gene expression mapped to *S. wiggsiae* and *Dialister invisus* in dentin compared with coronal caries. Dentin caries had greater number of over-represented activities from GO terms compared with health or coronal caries. Dentin caries had a high number of GO terms associated with sugar metabolism.

We conclude that in the samples analyzed, dentin caries appeared more active than coronal lesions with greater sugar metabolism and that *S. wiggsiae* was a major player in lesions advanced into dentin.

